# Supplementary material for: Mapping the evolution of fertility support policies in China: A content and instrumental analysis
Source: PLoS One. 2025 Oct 9;20(10):e0332137. doi: 10.1371/journal.pone.0332137 (PMC12510515; doi:10.1371/journal.pone.0332137)
Supplement: S1 Appendix — (ZIP) [file pone.0332137.s001.zip › S1 Appendix. 226 original policy documents/21-国家卫生计生委办公厅关于开展计划生育家庭科学育儿和青少年健康发展试点工作的通知(FBM-CLI-4-247391).doc]

国家卫生计生委办公厅关于开展计划生育家庭科学育儿和青少年健康发展试点工作的通知

**发布部门：** [国家卫生和计划生育委员会(已撤销)](https://www.pkulaw.com/law/chl?Aggs.IssueDepartment=60319) [机构沿革](javascript:void(0))

**发文字号：**国卫办家庭函〔2015〕344号

**发布日期：**2015.04.22

**实施日期：**2015.04.22

**时效性：** [现行有效](https://www.pkulaw.com/law/chl?Aggs.TimelinessDic=01)

**效力级别：** [部门工作文件](https://www.pkulaw.com/law/chl?Aggs.EffectivenessDic=XE0303)

**法规类别：** [计划生育管理](https://www.pkulaw.com/law/chl?Aggs.Category=09705)

国家卫生计生委办公厅关于开展计划生育家庭科学育儿和青少年健康发展试点工作的通知
（国卫办家庭函〔2015〕344号）

各省、自治区、直辖市、计划单列市及新疆生产建设兵团卫生计生委（人口计生委），解放军、武警部队计划生育领导小组办公室，中直机关、中央国家机关人口计生委：
　　为做好计划生育家庭未成年人的社会行为教育和培养工作，我委决定在全国开展计划生育家庭科学育儿和青少年健康发展试点工作。具体事项如下：
　　**一、**总体要求
　　紧密结合卫生计生中心工作，以计划生育家庭需求为导向，按照“政府主导、社会参与、面向家庭、服务家长”的原则，组织动员各方力量，探索建立统筹协调工作机制，为广大计划生育家庭提供科学育儿和青少年健康发展指导服务，提升广大家长科学抚育婴幼儿和培养青少年健康成长的能力和水平，促进家庭发展与社会和谐。

　　**二、**工作目标
　　（一）全面了解试点地区计划生育家庭在育儿和青少年健康发展方面的需求，为制定相关家庭发展政策提供依据，试点范围内服务建档率达到60%以上。
　　（二）试点地区家长对育儿知识和技能的掌握水平逐年提高，为婴幼儿健康成长营造良好的家庭环境。家长接受育儿培训覆盖率达到80%以上。
　　（三）试点地区家庭成员，包括青少年本人对青春期生理和心理知识的知晓程度逐年提高，青少年友好的家庭环境逐渐形成。家庭成员接受培训或咨询覆盖率达80%以上。
　　（四）试点地区家庭成员在育儿、青少年培养方面接受服务满意率达70%以上。

　　**三、**主要任务
　　（一）了解需求，明确重点任务。 试点单位要组织开展面向计划生育家庭婴幼儿和青少年的服务需求摸底调查，建立服务档案，研究分析需求重点，结合卫生计生系统网络技术优势，组织制定切实可行的工作方案，明确重点任务、进度安排、实施方法和保障措施等。
　　（二）宣传普及核心信息和科学知识。要充分利用广播、电视、报刊、网站以及手机信息终端等媒体，普及家庭科学育儿、青少年健康发展的核心信息和科学知识，倡导科学理念，提高家庭对科学育儿和青少年健康发展重要性的认知程度和知识技能的掌握水平。组织开展多种形式的社会宣传活动，为家庭科学育儿和青少年健康发展营造良好的社会氛围。
　　（三）组织开展技能培训和上门服务。各试点单位要因地制宜，充分依托妇幼健康服务等网络，以国家卫生计生委编印的儿童早期发展系列教材及青少年相关教材为依据，以“创建幸福家庭活动”和“新家庭计划”培训内容为指导，围绕计划生育家庭在育儿、培养未成年人方面的现实需求，为广大家长提供指导培训及服务。根据国家基本公共卫生服务技术规范要求，开展上门指导服务，加强与家长的联系和沟通，提供科学、准确、适用的知识、信息及服务。
　　（四）鼓励社会力量参与服务。支持和引导社会组织开展科学育儿和青少年健康发展相关服务。鼓励政府购买服务，支持多种主体参与，为家庭提供科学养育、亲子活动、健康行为和生活方式培养、养生保健、儿童安全与应急避险、青春期保健和性健康知识等指导和服务。健全行业管理制度，规范相关服务内容。

　　**四、**工作进度
　　2015年4月，印发试点通知，明确试点单位（详见附件）。
　　2015年5-8月，各试点单位开展摸底调研，制定试点工作方案和年度工作计划，启动试点工作。
　　2015年9月-2016年，试点单位结合本地实际，组织开展试点培训及服务工作，探索模式经验，查找问题和不足，不断完善工作措施，确保试点工作有效开展。
　　2017年-2020年，在总结试点经验的基础上，分地区分类指导，稳步推进，不断扩大试点，逐步实现家庭科学育儿和青少年健康发展工作的全面开展。

　　**五、**工作要求
　　（一）各级卫生计生部门要高度重视，将这两项工作纳入卫生计生中心工作总体规划，明确目标任务，同部署同落实。要保障经费投入，设立专项资金，在人员、设备等方面予以支持，保障工作深入持续开展。省级卫生计生部门要加强对本地区试点单位的调研指导，在省内推广成功经验，切实发挥其引领和带头作用。
　　（二）各级卫生计生部门承担试点工作的组织协调任务，要加强与相关部门和系统内相关单位的沟通联系，各司其职，加强协作，探索建立跨部门工作机制，共同推动试点工作的全面落实。
　　（三）国家卫生计生委将对各试点单位工作情况进行评估和督导，重点评估试点工作组织、管理情况及效果。省级卫生计生部门要对本地试点工作开展情况进行定期检查和年度评估，及时发现问题并协调解决，确保试点工作落实到位，并及时将情况报送我委。
　　联系人：国家卫生计生委家庭司　徐　航、郑春梅
　　联系电话：010-62030764/865、010-62030874（传真）
　　附件：1.计划生育家庭科学育儿试点单位名单
　　2.计划生育家庭青少年健康发展试点单位名单

国家卫生计生委办公厅
2015年4月22日

　　计划生育家庭科学育儿试点单位名单

　　1.北京市东城区
　　2.北京市平谷区
　　3.天津市和平区
　　4.天津市西青区
　　5.河北省石家庄市新华区
　　6.河北省石家庄市藁城市
　　7.山西省太原市杏花岭区
　　8.山西省运城市河津市
　　9.山西省长治市
　　10.内蒙古自治区呼和浩特市新城区
　　11.内蒙古自治区包头市昆区
　　12.内蒙古自治区锡林郭勒盟乌拉盖管理区
　　13.辽宁省沈阳市沈河区
　　14.辽宁省沈阳市大东区
　　15.辽宁省朝阳市龙城区
　　16.辽宁省锦州市北镇市
　　17.吉林省四平市铁西区
　　18.黑龙江省大兴安岭地区
　　19.黑龙江省牡丹江市东宁县
　　20.上海市徐汇区
　　21.上海市松江区
　　22.江苏省常州市天宁区
　　23.江苏省苏州市常熟市
　　24.浙江省杭州市下城区
　　25.浙江省嘉兴市嘉善县
　　26.安徽省芜湖市镜湖区
　　27.安徽省安庆市太湖县
　　28.福建省福州市鼓楼区
　　29.福建省三明市泰宁县
　　30.江西省九江市浔阳区
　　31.江西省上饶市万年县
　　32.山东省济南市市中区
　　33.山东省潍坊市诸城市
　　34.河南省郑州市中原区
　　35.河南省安阳市林州市
　　36.湖北省武汉市武昌区
　　37.湖北省黄冈市蕲春县
　　38.湖南省长沙市芙蓉区
　　39.湖南省衡阳市雁峰区
　　40.广东省广州市海珠区
　　41.广东省深圳市宝安区
　　42.广东省惠州市惠东县
　　43.广西壮族自治区桂林市七星区
　　44.广西壮族自治区贺州市钟山县
　　45.海南省海口市秀英区
　　46.海南省澄迈县
　　47.重庆市江北区
　　48.重庆市铜梁县
　　49.四川省成都市青羊区
　　50.四川省德阳市
　　51.贵州省贵阳市乌当区
　　52.贵州省黔南州荔波县
　　53.云南省昆明市官渡区
　　54.云南省大理州宾川县
　　55.西藏自治区拉萨市城关区
　　56.陕西省渭南市韩城市
　　57.甘肃省嘉峪关市
　　58.甘肃省天水市秦州区
　　59.青海省西宁市城中区
　　60.青海省海东市平安县
　　61.宁夏回族自治区银川市金凤区
　　62.宁夏回族自治区石嘴山市平罗县
　　63.新疆维吾尔自治区克拉玛依市独山子区
　　64.新疆维吾尔自治区昌吉州昌吉市

　　计划生育家庭青少年健康发展试点单位名单

　　1. 北京市海淀区
　　2. 北京市怀柔区
　　3. 天津市河西区
　　4. 天津市滨海新区
　　5. 河北省石家庄市裕华区
　　6. 河北省石家庄市赞皇县
　　7. 山西省阳泉矿区
　　8. 山西省长治市长治县
　　9. 山西省临汾市永和县
　　10. 内蒙古自治区乌海市
　　11. 内蒙古自治区伊金霍洛旗
　　12. 辽宁省营口市站前区
　　13. 辽宁省大连市普兰店市
　　14. 辽宁省大连市金州区
　　15.吉林省延边朝鲜族自治州敦化市
　　16.黑龙江省哈尔滨市南岗区
　　17.黑龙江省哈尔滨市尚志市
　　18.上海市杨浦区
　　19.上海市金山区
　　20.江苏省南京市栖霞区
　　21.江苏省无锡市江阴市
　　22.浙江省杭州市西湖区
　　23.浙江省嘉兴市海宁市
　　24.安徽省合肥市蜀山区
　　25.安徽省滁州市天长市
　　26.福建省泉州晋江市
　　27.福建省漳州龙海市
　　28.江西省新余市渝水区
　　29.江西省九江市德安县
　　30.山东省青岛市经济技术开发区
　　31.山东省烟台市莱州市
　　32.河南省郑州市惠济区
　　33.河南省焦作市孟州市
　　34.河南省焦作市沁阳市
　　35.湖北省武汉市洪山区
　　36.湖北省荆门市钟祥市
　　37.湖南省娄底市
　　38.广东省广州市海珠区
　　39.广东省深圳市宝安区
　　40.广东省惠州市惠东县
　　41.广西壮族自治区南宁市青秀区
　　42.广西壮族自治区来宾市武宣县
　　43.海南省海口市龙华区
　　44.海南省昌江县
　　45.重庆市渝中区
　　46.重庆市永川区
　　47.四川省成都市成华区
　　48.四川省成都市崇州市
　　49.贵州省安顺市西秀区
　　50.贵州省黔东南州岑巩县
　　51.云南省红河州蒙自市
　　52.云南省玉溪市新平县
　　53.西藏自治区拉萨市城关区
　　54.陕西省西安市未央区
　　55.甘肃省兰州市安宁区
　　56.甘肃省兰州市榆中县
　　57.青海省海西州格尔木市
　　58.青海省西宁市湟中县
　　59.宁夏回族自治区银川市西夏区
　　60.宁夏回族自治区吴忠市盐池县
　　61.新疆维吾尔自治区克拉玛依市克拉玛依区
　　62.新疆维吾尔自治区昌吉州阜康市

© 北大法宝：（[www.pkulaw.com](https://www.pkulaw.com/)) 专业提供法律信息、法学知识和法律软件领域各类解决方案。北大法宝为您提供丰富的参考资料，正式引用法规条文时请与标准文本核对。
欢迎查看所有[产品和服务](https://www.pkulaw.com/helps/2.html)。法宝快讯：[如何快速找到您需要的检索结果？](https://www.pkulaw.com/helps/70.html)[法宝](https://www.pkulaw.com/helps/69.html)V6有何新特色？

扫描二维码阅读原文

**原文链接：**<https://www.pkulaw.com/chl/03856923f9a27c15bdfb.html>
